# Genomic prediction of tuberculosis drug-resistance: benchmarking existing databases and prediction algorithms

**DOI:** 10.1186/s12859-019-2658-z

**Published:** 2019-02-08

**Authors:** Tra-My Ngo, Yik-Ying Teo

**Affiliations:** 1 NUS Graduate School for Integrative Science and Engineering, National University of Singapore, Singapore, 119077 Singapore; 2 Saw Swee Hock School of Public Health, National University of Singapore, 12 Science Drive 2, Singapore, 117549 Singapore; 3 Department of Statistics and Applied Probability, National University of Singapore, Singapore, Singapore; 4 Life Sciences Institute, National University of Singapore, Singapore, 117456 Singapore; 5 Genome Institute of Singapore, Agency for Science, Technology and Research, Singapore, 138672 Singapore

**Keywords:** Tuberculosis, Drug-resistance, Genomic prediction

## Abstract

**Background:**

It is possible to predict whether a tuberculosis (TB) patient will fail to respond to specific antibiotics by sequencing the genome of the infecting *Mycobacterium tuberculosis* (*Mtb*) and observing whether the pathogen carries specific mutations at drug-resistance sites. This advancement has led to the collation of TB databases such as PATRIC and ReSeqTB that possess both whole genome sequences and drug resistance phenotypes of infecting *Mtb* isolates. Bioinformatics tools have also been developed to predict drug resistance from whole genome sequencing (WGS) data.

Here, we evaluate the performance of four popular tools (TBProfiler, MyKrobe, KvarQ, PhyResSE) with 6746 isolates compiled from publicly available databases, and subsequently identify highly probable phenotyping errors in the databases by genetically predicting the drug phenotypes using all four software.

**Results:**

Our results show that these bioinformatics tools generally perform well in predicting the resistance status for two key first-line agents (isoniazid, rifampicin), but the accuracy is lower for second-line injectables and fluoroquinolones. The error rates in the databases are also non-trivial, reaching as high as 31.1% for prothionamide, and that phenotypes from ReSeqTB are more susceptible to errors.

**Conclusions:**

The good performance of the automated software for drug resistance prediction from TB WGS data shown in this study further substantiates the usefulness and promise of utilising genetic data to accurately profile TB drug resistance, thereby reducing misdiagnoses arising from error-prone culture-based drug susceptibility testing.

**Electronic supplementary material:**

The online version of this article (10.1186/s12859-019-2658-z) contains supplementary material, which is available to authorized users.

## Background

While the prevalence of tuberculosis (TB) worldwide has been decreasing, the emergence of drug-resistance forms of TB (DR-TB) poses a new public health crisis in numerous parts of the world, with multidrug-resistant TB (MDR-TB) alone being responsible for 480,000 deaths annually [1]. The World Health Organisation (WHO) has recommended that all TB patients should be tested for drug resistance [2], although conventional drug susceptibility testing (DST) can take more than 6 weeks and requires a laboratory equipped for strict biosafety [3]. These infrastructural and time requirements mean the majority of treatment-seeking TB patients in lower- and middle-income countries are started on first-line TB drugs regardless of their drug-resistance status, as there are inadequate resources and capacity to screen every TB patient [1].

Drug resistance fundamentally develops through the accrual of specific mutations in the genome of the infecting pathogen *Mycobacterium tuberculosis* (*Mtb*) [4]. By performing whole genome sequencing (WGS) of infecting *Mtb* isolates which have undergone conventional DST, the mutations that confer drug resistance to specific antibiotics can be identified [5]. Sufficiently large databases possessing both WGS and DST information have allowed the drug resistance to several antibiotics to be accurately predicted, to the extent that rapid diagnostics that target these mutations have been developed [6–8]. GeneXpert MTB/RIF is one of these rapid diagnostic tests that predicts rifampicin resistance by querying specific positions in the *Mtb* genome [9].

One of the key advantages these diagnostics possess over conventional DST is the speed at which the drug resistance profile can be obtained. For example, GeneXpert MTB/RIF can detect MTB and the genetic determinants of RR-TB in 2 h [10], and this allows the timely identification of optimal treatment regimens. The premise is simple: if a patient is diagnosed early to suffer from a form of TB that is resistant to rifampicin, the physician will prescribe an alternate rifampicin-free regimen in order to ensure efficacious treatment, instead of maintaining a standard but ineffective first-line regimen for 6 months.

While the premise is straightforward, the majority of present diagnostics are however confined to screening for resistance to a limited range of antibiotics. What this means is that a positive result from GeneXpert may indicate rifampicin resistance, but does not provide any insights towards the resistance profiles to other anti-TB agents. Physicians escalate to second-line agents except this is again unsupported by whether the patient is resistant to any of the second-line agents.

The ability for WGS to simultaneously infer the resistance profiles to numerous anti-TB agents is thus attractive, since the physician can be guided to prescribe a combination of antibiotics that is more likely to be effective. Operationally, this relies on having a well-curated knowledge of the range of genetic mutations that confer resistance to the different anti-TB agents, as well as bioinformatic tools that translate the genomic sequence to drug resistance information that can be understood by physicians.

Several databases such as PATRIC [11–13] and ReSeqTB [14, 15] possessed both the genomic sequences and DST phenotypes of thousands of infecting *Mtb* isolates from different parts of the world, and these have facilitated the identification of genomic sites associated with drug-resistance. Drug-resistance prediction software such as TBProfiler [16], MyKrobe [17]. KvarQ [18], and PhyResSE [19], rely on these genomic sites to predict the drug resistance profile of a *Mtb* genome to the range of anti-TB agents. Oftentimes, the phenotypes from conventional DST are assumed to be free from errors, and this can invariably confound the identification of resistance-conferring genomic sites especially for anti-TB drugs such as streptomycin, ethambutol and pyrazinamide where the DST error rates have been reported to be higher [3].

Here, we aim to benchmark the sensitivity and specificity of the four prediction software, and to evaluate the fidelity of the reported DST phenotypes in 6746 *Mtb* isolates compiled from the PATRIC [11–13] and ReSeqTB [14, 15] databases, and 10 other studies. The evaluation is achieved by comparing the reported phenotypes against the genetically inferred phenotypes, and this consequently allowed the identification of the anti-TB drugs where laboratory-determined DST results are more likely to be erroneous.

## Materials and methods

### Genome sequences and drug-susceptibility phenotypes for Mtb isolates

We focused on two databases hosting *Mycobacterium tuberculosis* (*Mtb*) isolates with complete genomic sequences and drug-susceptibility phenotypes: (1) PATRIC – which hosts nearly 150,000 genomes belonging to more than 20 bacterial genera with drug resistance status for nearly 100 antibiotics [12, 13]; and (2) ReSeqTB – which is a specific database for driving the development of novel rapid diagnostic tests and personalised treatment of TB [14, 15]. The drugs that our study considered include five first-line agents: rifampicin (RIF), isoniazid (INH), pyrazinamide (PZA), ethambutol (EMB), streptomycin (STM); 3 second-line injectables: amikacin (AMK), capreomycin (CAP), kanamycin (KAN); three oral second-line drugs: ethionamide (ETO), prothionamide (PTO), p-aminosalicyclic acid (PAS); and three fluoroquinolones: ciprofloxacin (CIP), moxifloxacin (MFX), ofloxacin (OFX).

Data from PATRIC was obtained by querying all *Mtb* contributions with resistance profiles for anti-TB drugs, and whose genome sequences were also contributed to the Sequencing Reads Archive (SRA) and BioProject accessions (accessed Jan 5, 2018). This yielded a set of 5035 isolates. For ReSeqTB, we considered the samples available from the repository in the folder Databases/ReSeqTB/fullExportDb-1254-External-CSV/msf.xls (accessed Jan 18, 2018) with available SRA accessions, which yielded a set of 3568 isolates.

In addition, we included another set of 5471 isolates identified from a literature review by querying PUBMED for “whole genome sequencing drug resistant tuberculosis”, retaining only samples with both whole genome sequences and conventional DST phenotypes that are available online publicly and conforming to the following inclusion criteria: (1) isolates should be from clinical samples; (2) there should be at least 10 samples from each study; (3) DST must be performed on at least four of the five first-line drugs; and (4) the study must possess at least one drug-resistant TB isolate (see Additional file 1). This set is subsequently referred to as the “LitRev” set.

For the three datasets that we considered, comprising of PATRIC, ReSeqTB, and LitRev, we highlight there are samples that are found in more than one dataset, including with conflicting DST results for specific drugs. Further details on harmonising the datasets for our analyses can be found in the next section.

### Data quality control

The mapping quality of the genome sequences for all samples were assessed to identify samples with poor quality sequencing, as well as samples that were incorrectly classified as *Mtb* (see Additional file 2). There are samples with multiple DST outcomes for a specific drug, either due to the sample being located in more than one dataset, or because there are multiple DST results from within one dataset. For these samples, we retained only one final DST outcome according to the following criteria: (i) if the multiple DST outcomes are consistent, then assigning the final outcome is trivial; (ii) if the sample is located in more than one dataset and there is a discordance in the multiple DST outcomes, we discard the outcomes from ReSeqTB and/or PATRIC, and either assign the outcome from LitRev or discard the sample entirely; (iii) if the sample belongs uniquely to PATRIC and contains multiple outcomes, the results obtained from an WHO-endorsed protocol were retained.

### Bioinformatics tools for inferring drug-resistance

We considered four bioinformatics software that are designed to infer the resistance profiles of anti-TB drugs using the *Mtb* genome sequence: TBProfiler [16], MyKrobe [17], KVarQ [18], and PhyResSE [19]. All four software take in raw WGS data such as fastq or bam files, and produce results in varying interfaces that are specifically designed to aid interpretation by clinical microbiologists. These four software predict resistance to all five first-line drugs and most of the key second-line agents, by considering the genetic alleles on different panels of curated mutations that are associated with drug resistance (see Additional file 3). The different software utilise different bioinformatics algorithms and may refer to a dissimilar panel of mutations to predict resistance, thereby producing outcomes that may differ between the four tools. Specifically, TBProfiler maps input sequence to a truncated version of the H37Rv reference genome (GenBank accession number: NC_000962.3), which contains only the regions of interest to drug resistance prediction, before identifying the presence of genetic markers of resistance. On the other hand, MyKrobe compares the de Bruijn graph of the input sequence with the graph built from a collection of resistant and susceptible alleles on diverse genetic backgrounds to determine resistance profile. In KVarQ, each read from the input is queried against a series of target sequences constructed from known mutations or regions associated with drug resistance, with each match increasing the confidence of the presence of the genetic markers in the sample. In contrast, PhyResSE follows a more traditional route of mapping the fastq input to the complete reference genome and then calling all single nucleotide polymorphisms (SNPs) and small indels before comparing them to its mutations panel. All four tools however allow the panel of mutations to be updated whenever new markers of drug resistance are discovered, and allow the inference of the lineage of the *Mtb* samples. Among the four tools, MyKrobe possesses the additional capability to identify species in situations of mixed infections, while PhyResSE additionally allows stringent pre-processing and quality control of sequencing data. In our evaluation, we used TBProfiler version 0.3.2, MyKrobe v0.5.6–0-gbd7923a-dirty, KvarQ version 0.12.3a1, and PhyResSE pipeline version metaphyresse.v7 implemented locally with SNP catalogue version 29.

### Calculating sensitivity and specificity of bioinformatics tools and DST credibility of the samples

All sequencing reads sets were used as inputs to each of the TB resistance prediction algorithms. In case of no calls or if there were discordant results among the reads sets of a sample for a drug, the prediction for the sample for that particular drug was treated as missing. In our sensitivity and specificity calculation and parameter estimation for DST credibility computation, which are subsequently described in detail, we omitted those samples clearly indicated to have been used to train the tools. The sensitivities and specificities of the four algorithms for each drug were first calculated together with their 95% confidence interval, with the corresponding phenotypes in the data collection as the gold standard, except for the case of fluoroquinolones, where the performance was benchmarked to CIP, MFX, and OFX individually; the case of PTO, where the performance was benchmarked against ETO; and the case of KVarQ’s Kanamycin/Amikacin prediction, which was benchmarked against KAN and AMK individually. The DST credibility score for each sample was calculated as the probability that the phenotype of the sample is correctly specified given the prediction results from the four programs. Specifically, for a drug *i*, let the prevalence of drug resistance be *P*_*i*_, and the true sensitivity, the true specificity, the proportion of no-call predictions among truly resistant samples and the proportion of no-call predictions among truly susceptible samples of each program be *Sens*_*ij*_, *Spec*_*ij*_, *NoR*_*ij*_, and *NoS*_*ij*_, for *j* in {*TBProfiler*, *KVarQ*, *MyKrobe*, *PhyResSE*}. We denote the predictions of the four algorithms as

$$ predictions=\left\{{Y}_j\right|\ j\in \left\{ TBProfiler, KVarQ, MyKrobe, PhyResSE\right\}, $$

where *Y*_*j*_ = 1 if the sample is predicted resistant, 0 if susceptible, and NA otherwise. The probability of the sample being resistant given the predictions of the four tools is as follows:

$$ P\left( sample\ is\ resistant\ \right|\  predictions\Big)=\frac{P\left( predictions\right| sample\ is\ resistant\Big).{P}_i}{Likelihood(predictions)}, $$

with *Likelihood*(*predictions*) calculated as

$$ P\left( predictions\right| sample\ is\ resistant\left).{P}_i+P\left( predictions\right| sample\ is\ susceptible\right).\left(1-{P}_i\right)=\left[{\prod}_jP\left({Y}_j\right| sample\ is\ resistant\Big)\right].{P}_i+\left[{\prod}_jP\left({Y}_j\right| sample\ is\ susceptible\Big)\right].\left(1-{P}_i\right), $$

where

$$ P\left({Y}_j\right| sample\ is\ resistant\Big)=\left\{\begin{array}{c} Sen{s}_{ij}\  if\ {Y}_j=1\\ {}1- Sen{s}_{ij}- No{R}_{ij}\  if\ {Y}_j=0\\ {} No{R}_{ij}\  if\ {Y}_j\  is\  NA\end{array}\right., $$

and

$$ P\left({Y}_j\right| sample\ is\ susceptible\Big)=\left\{\begin{array}{c}1- Spe{c}_{ij}- No{S}_{ij}\  if\ {Y}_j=1\\ {} Spe{c}_{ij}\  if\ {Y}_j=0\\ {} No{S}_{ij}\  if\ {Y}_j\  is\  NA\ \end{array}\right.. $$

The DST credibility score of a sample thus equals *P*(*sample is resistant* | *predictions*) if its reported phenotype is resistant, or 1 – *P*(*sample is resistant* | *predictions*) if the reported phenotype is susceptible. The quantities *P*_*i*_, *Sens*_*ij*_, *Spec*_*ij*_, *NoR*_*ij*_, and *NoS*_*ij*_ were maximum likelihood estimates (MLE) obtained from an EM algorithm. We chose to use the MLE estimates, as opposed to incorporate informative prior distributions and obtain maximum a posteriori (MAP) estimates, due to the lack of prior knowledge to select meaningful prior distribution for these parameters. The EM algorithm was initialized with the set of parameters derived by assuming the conventional DST results for the samples were impeccable. The sensitivity and specificity estimates obtained from this model are subsequently called the adjusted sensitivity and specificity. Proxy for the rate of misclassification of phenotypic DST results for each drug in a database, either individual or merged database, is calculated as the proportion of samples with DST credibility score smaller than 0.5 to the total number of valid samples. Proxy for the rate of non-utilizable data for a drug is calculated as the proportion of samples removed from the database due to discordant phenotypic results to the total number of samples with good mapping quality WGS data and drug resistance results for that drug in the database.

## Results

The total number of samples across the three databases with both WGS and drug resistance phenotypes for at least one drug is 6756, of which 2302 samples are present in all three databases (see Additional file 4: Figure S1, Additional file 5). After excluding samples with poor sequencing quality (10 samples, see Additional file 2) and discordant DST phenotypes, the number of unique isolates ranges from 4831 to 6694 for first-line agents (STM, RIF respectively), and from 457 to 2424 for second-line and third-line agents (CIP, OFX respectively, see Additional file 6). After excluding samples used in the training of the software, the figure ranges from 4357 to 5026 for first-line agents (STM, PZA respectively) and from 191 to 2424 for second- and third-line agents (CIP and OFX respectively, see Table 1).

**Table 1:** Empirical sensitivities and specificities of four software for predicting anti-TB drug resistance

Drug	Number of samples	Sensitivity				Specificity			
		TBProfiler	MyKrobe	KVarQ	PhyResSE	TBProfiler	MyKrobe	KVarQ	PhyResSE
INH	4840	0.92 (0.91, 0.93)	0.91 (0.89, 0.92)	0.89 (0.88, 0.90)	0.91 (0.89, 0.92)	0.94 (0.93, 0.95)	0.98 (0.97, 0.98)	0.98 (0.98, 0.99)	0.97 (0.96, 0.98)
RIF	4843	0.91 (0.89, 0.92)	0.92 (0.91, 0.94)	0.92 (0.90, 0.93)	0.94 (0.93, 0.95)	0.95 (0.94, 0.96)	0.97 (0.96, 0.97)	0.97 (0.96, 0.97)	0.96 (0.95, 0.97)
EMB	4585	0.91 (0.89, 0.92)	0.83 (0.81, 0.86)	0.65 (0.62, 0.67)	0.76 (0.73, 0.78)	0.83 (0.81, 0.84)	0.86 (0.85, 0.87)	0.91 (0.90, 0.92)	0.88 (0.87, 0.89)
PZA	5026	0.59 (0.55, 0.63)	0.38 (0.34, 0.42)	0.54 (0.50, 0.58)	0.58 (0.54, 0.61)	0.92 (0.91, 0.93)	0.98 (0.98, 0.99)	0.94 (0.93, 0.94)	0.97 (0.97, 0.98)
STM	4357	0.82 (0.80, 0.84)	0.79 (0.77, 0.81)	0.75 (0.73, 0.77)	0.76 (0.74, 0.78)	0.86 (0.85, 0.87)	0.93 (0.92, 0.94)	0.92 (0.91, 0.93)	0.92 (0.91, 0.93)
AMK	1649	0.90 (0.86, 0.93)	0.75 (0.70, 0.80)	0.75 (0.69, 0.79)	0.79 (0.74, 0.83)	0.75 (0.73, 0.78)	0.99 (0.98, 1.00)	0.99 (0.98, 0.99)	0.99 (0.98, 0.99)
CAP	1830	0.71 (0.66, 0.76)	0.67 (0.62, 0.73)	NA	0.71 (0.65, 0.75)	0.95 (0.93, 0.96)	0.93 (0.92, 0.95)	NA	0.96 (0.94, 0.97)
KAN	1578	0.87 (0.83, 0.90)	0.75 (0.71, 0.80)	0.72 (0.67, 0.76)	0.82 (0.77, 0.86)	0.96 (0.94, 0.97)	0.98 (0.97, 0.99)	0.99 (0.98, 0.99)	0.97 (0.96, 0.98)
CIP	191	0.87 (0.75, 0.94)	0.83 (0.71, 0.92)	0.82 (0.70, 0.90)	0.88 (0.77, 0.95)	0.97 (0.92, 0.99)	0.98 (0.93, 1.00)	0.97 (0.92, 0.99)	0.98 (0.93, 1.00)
MFX	1086	0.68 (0.61, 0.75)	0.61 (0.53, 0.68)	0.58 (0.50, 0.65)	0.67 (0.60, 0.74)	0.93 (0.91, 0.94)	0.95 (0.94, 0.97)	0.93 (0.91, 0.94)	0.95 (0.93, 0.96)
OFX	2424	0.81 (0.78, 0.84)	0.74 (0.71, 0.78)	0.72 (0.68, 0.75)	0.81 (0.77, 0.84)	0.96 (0.95, 0.97)	0.98 (0.97, 0.98)	0.96 (0.95, 0.97)	0.97 (0.97, 0.98)
ETO	835	0.41 (0.35, 0.48)	NA	NA	0.07 (0.04, 0.11)	0.82 (0.79, 0.85)	NA	NA	0.97 (0.95, 0.98)
PTO	540	0.30 (0.24, 0.37)	NA	NA	0.02 (0.01, 0.05)	0.92 (0.89, 0.95)	NA	NA	1.00 (0.98, 1.00)
PAS	469	0.14 (0.08, 0.24)	NA	NA	0.00 (0.00, 0.04)	0.97 (0.95, 0.98)	NA	NA	1.00 (0.99, 1.00)

Numbers in brackets represent the corresponding 95% confidence intervals. An NA is assigned when the software does not predict the resistance profile for the specific drug

### Benchmarking performance of drug-resistance inference software

We considered four bioinformatics software (TBProfiler, KVarQ, MyKrobe, PhyResSE) capable of inferring resistance to a spectrum of antibiotics based on the genomic sequence of the infecting *Mtb* agent. For each software, we calculated the empirical sensitivity and specificity using the isolates from the databases by comparing the genetically-inferred phenotypes for each drug against the reported DST phenotypes, under the assumption that the latter are accurate.

We observed that the sensitivity of TBProfiler was consistently higher than those of the other three predicting software for the majority of the 14 drugs considered (see Table 1). However this came at a marginal compromise on the specificity, which was lower for TBProfiler across most of the drugs when compared to the other three software. For example, in the case of amikacin (AMK) with data from 1649 isolates, the sensitivity from TBProfiler was the highest at 90% whereas those from MyKrobe, KVarQ and PhyResSE were at 75, 75 and 79% respectively; conversely, the specificity of TBProfiler was the lowest at 75% whereas those from the other three software were at 99%. Overall, there were specific drugs whose resistance profiles (whether susceptible or resistant) were better predicted by each software.

At the drug-level, the common first-line agents such as isoniazid (INH) and RIF can be predicted with sensitivities and specificities in excess of 90% by all four software; whereas the resistance status for antibiotics such as pyrazinamide (PZA), ethionamide (ETO), prothionamide (PTO), and para-aminosalicylic acid (PAS) reported sensitivities lower than 60%.

### Identifying misspecified DST results and re-evaluating software performance

We have assumed the reported DST phenotypes in the public databases are accurate in assessing the performance of the predicting software. However, laboratory-ascertained DST results can contain errors due to the semi-qualitative nature of the assessment. As such, we used a combination of all four predictors to probabilistically assess the likelihood that each reported phenotype is an error, after accounting for the degree of confidence in ascertaining the accuracy of the genetically inferred phenotype.

**Fig. 1:**
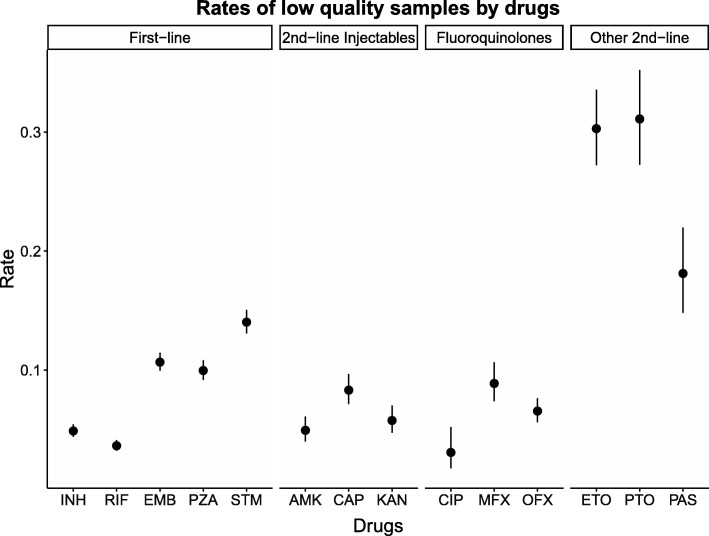
Misclassification rates of laboratory-based DST results by drugs. The solid circles represent the point estimates of the misclassification rates upon comparing the laboratory-based DST phenotypes with genetically-inferred drug-resistant phenotypes. The genetically-inferred phenotypes were probabilistically ascertained using all four software. The vertical lines represent the corresponding 95% confidence intervals

We observed the error rates were less than 5% for CIP, RIF, INH and AMK, although two of the remaining three first-line agents (EMB, STM) yielded error rates in excess of 10% (see Fig. 1, Additional file 6). The rates of misclassification were considerably higher for the second-line agents such as ETO, PTO and PAS, although the uncertainty of these estimates were considerably larger given the smaller sample sizes and poorer sensitivities of the software in inferring isolates with genuine resistance to these drugs.

In assessing the errors, we also evaluated which database was more prone to erroneous phenotypes. We observed similar trends across the three databases (PATRIC, ReSeqTB, LitRev) for most of the drugs except for PAS, where the misclassification rate was considerably lower at 2.6% in ReSeqTB compared to 20.8% in PATRIC and 10.8% in LitRev (see Table 2). However, we observed that the rate of non-utilizable data was generally highest in ReSeqTB (except for KAN, CIP, ETO and PAS).

**Table 2:** Summary of isolates in the three TB databases according to the 14 anti-TB drugs

Drug	PATRIC				ReSeqTB				LitRev			
	*N*_*clean*_	*R*_*error*_	*N*_*full*_	*R*_*missing*_	*N*_*clean*_	*R*_*error*_	*N*_*full*_	*R*_*missing*_	*N*_*clean*_	*R*_*error*_	*N*_*full*_	*R*_*missing*_
INH	5018	0.043	5018	0.000	3503	0.045	3555	0.015	5451	0.043	5451	0.000
RIF	4979	0.028	4981	0.000	3459	0.038	3521	0.018	5416	0.034	5416	0.000
EMB	4739	0.075	4787	0.010	3497	0.098	3546	0.014	5335	0.092	5335	0.000
PZA	3633	0.054	3633	0.000	3298	0.095	3346	0.014	4752	0.082	4752	0.000
STM	3367	0.141	3380	0.004	1951	0.129	2008	0.028	3716	0.129	3716	0.000
AMK	1131	0.034	1131	0.000	983	0.040	993	0.010	1256	0.037	1256	0.000
CAP	1100	0.055	1101	0.001	1158	0.076	1167	0.008	1587	0.059	1588	0.001
KAN	1348	0.056	1350	0.001	716	0.031	716	0.000	1095	0.031	1095	0.000
CIP	340	0.018	340	0.000	358	0.031	358	0.000	313	0.016	313	0.000
MFX	726	0.059	726	0.000	874	0.071	885	0.012	993	0.057	993	0.000
OFX	836	0.065	851	0.018	1163	0.070	1188	0.021	1818	0.052	1818	0.000
ETO	559	0.370	562	0.005	252	0.159	252	0.000	321	0.265	321	0.000
PTO	52	0.115	52	0.000	410	0.337	431	0.049	498	0.301	498	0.000
PAS	375	0.208	375	0.000	78	0.026	78	0.000	74	0.108	74	0.000

*N*_*clean*_ refers to the number of isolates with valid DST results and genetically-inferred credibility scoring; *R*_*error*_ refers to the misclassification rate in each database, defined as the proportion of the *N*_*clean*_ isolates with DST credibility scores < 0.5; *N*_*full*_ is defined as the summation of *N*_*clean*_ and the number of good mapping quality isolates with no genetically-inferred credibility scoring; and *R*_*missing*_ refers to the proportion of *N*_*full*_ isolates that presented unusable laboratory-based DST phenotypes due to either inconsistent results (across multiple DST phenotype entries for the same isolate) or documentation errors across the databases

While inferring the likely erroneous laboratory-based DST phenotypes by their genetically-inferred phenotypes, we also obtained a calibrated performance of the four software as part of the model output. In theory this provides a better indication of the performance of these software, except there is the possibility of overfitting (see Discussion later). In general, the estimated sensitivity and specificity figures were higher after adjusting the phenotypes (see Table 3), although surprisingly there were some decreases in sensitivity (PAS) and specificity (AMK, INH) for TBProfiler.

**Table 3:** Calibrated sensitivities and specificities of four software for predicting anti-TB drug resistance

Drug	Number of samples	Sensitivity				Specificity			
		TBProfiler	MyKrobe	KVarQ	PhyResSE	TBProfiler	MyKrobe	KVarQ	PhyResSE
INH	4840	0.99	0.99	0.99	1.00	0.94	0.99	1.00	0.99
RIF	4843	0.96	0.97	0.96	0.99	0.98	1.00	1.00	1.00
EMB	4585	0.99	0.93	0.69	0.84	0.96	1.00	1.00	0.99
PZA	5026	0.83	0.40	0.79	0.66	0.97	1.00	0.99	1.00
STM	4357	1.00	0.91	0.99	0.99	0.90	0.94	1.00	0.99
AMK	1649	1.00	0.96	0.97	1.00	0.75	1.00	1.00	0.99
CAP	1830	0.99	0.95	NA	0.99	0.99	0.98	NA	1.00
KAN	1578	1.00	0.89	0.84	1.00	0.96	1.00	1.00	1.00
CIP	191	0.95	0.95	0.93	1.00	0.98	1.00	0.99	1.00
MFX	1086	0.97	0.87	0.85	0.99	0.98	1.00	0.97	1.00
OFX	2424	0.97	0.89	0.87	0.99	0.98	1.00	0.98	1.00
ETO	835	0.95	NA	NA	0.21	0.94	NA	NA	1.00
PTO	540	0.80	NA	NA	0.05	0.99	NA	NA	1.00
PAS	469	0.14	NA	NA	0.00	0.97	NA	NA	1.00

An NA is assigned when the software does not predict the resistance profile for the specific drug

Despite the adjustment, the ability to predict the presence of drug-resistance remains poor for ETO, PAS, PTO and PZA. However, the recalibration indicated that the majority of the software were able to deliver sensitivity in excess of 90% for INH, RIF, EMB, STM, AMK, CAP, KAN, CIP, MFX, and OFX. The trends in the relative performance between the four software remained unchanged after the recalibration, with TBProfiler being the most sensitive software in the detection of resistance in most of the 14 drugs at the expense of marginally lower specificities compared to MyKrobe, KVarQ and PhyResSE.

## Discussion

The ability to rapidly identify which are the viable drugs for treating a TB infection is likely to be increasingly urgent, given the proliferation of drug-resistant strains of the *Mtb* pathogen. The use of whole-genome sequencing to replace conventional laboratory-based drug-susceptibility testing reduces the time taken to culture and test against individual drugs, but requires sophisticated bioinformatics algorithms to be designed and validated to translate the genetic information into drug susceptibility phenotypes. In this paper, we have evaluated four bioinformatic software for predicting TB drug resistance from *Mtb* genome sequences, using the largest set of isolates available and for 14 antibiotics. Using latent class modeling, we have identified which isolates in the existing databases are likely to present erroneous DST phenotypes and recalibrated the performance of these bioinformatics software adjusting for errors in the existing databases.

All four software present considerably accurate predictions for the majority of the 14 antibiotics, although the ability to correctly detect resistance tends to be lower than the ability to correctly detect the absence of resistance. These results concur with the findings of a previous comparison study [20], as well as that of a recent study where genetic sequencing was established to be sufficiently accurate for drug resistance surveillance in place of laboratory-based DST phenotyping [21]. However, we still observe low sensitivity in detecting resistance to ETO, PAS, PTO, and PZA. This could be attributed to the lack of information around which genetic polymorphisms are responsible in resisting the drugs, especially since the sample sizes for isolates with DST phenotypes at these drugs (except for PZA) are correspondingly lowest. Without a clear understanding to the genetic biomarkers of drug resistance or to their penetrance, or at least more powerful algorithms that can pick up additional intricacies of resistance across multiple drugs, such as what have been done for HIV and cancer [22–24], the ability to successfully predict the presence of drug-resistance is reduced. Thus, even though our inference on the accuracy of laboratory-based DST indicated there were higher rates of misclassification at these drugs, it should be highlighted that the estimation of the misclassification rates is itself subject to considerable errors.

Our evaluation of the performance of the bioinformatics predictors differed from previous reports, since we also calibrated the performance as we probabilistically determined the likely erroneous phenotype entries in the *Mtb* databases. These errors are adjusted to the (highly) probable correct phenotypes, inferred using the combined power of the four software with the assumption that the outputs of all four software are independent conditional on the genome sequence. This can, however, lead to the problem of over-fitting the recalibrated performance of the four software, as each software contributes partly to adjust the phenotypes with overlapping sets of genetic markers. For drugs that can be predicted with high levels of sensitivity and specificity, the likelihood of over-fitting is expected to be negligible. For drugs where either the sensitivity or specificity are comparatively lower, the recalibrated accuracy still serves as an upper bound to the performance of these software, while the unadjusted accuracy serves as the lower bound by calibrating the performance against a database with erroneous entries.

Our study has quantified the credibility of laboratory-based DST phenotypes for a large number of isolates with publicly available WGS data. The ability to perform in silico TB drug-resistance profiling provides the opportunity for a consistent, standardised, and accredited model to obtain DST phenotypes, one that is independent of variations in laboratory quality control. While there are still infrastructural limitations to the widespread adoption of WGS for identifying DR-TB in resource-poor settings, the availability of accurate bioinformatics predictors will undoubtedly be valuable for translating genetic sequences into clinically actionable information to guide efficacious drug prescription.

## Additional files

Additional file 1: Studies reviewed for the LitRev data set. This file presents the list of studies surveyed for LitRev. (XLSX 27 kb)

Additional file 2: Quality screening of sequencing data. This file details the quality control step of the study. (DOCX 26 kb)

Additional file 3: Characteristics of the four bioinformatics software for predicting drug resistance from *Mtb* genome sequences. This file delineates the features of the four tools TBProfiler, MyKrobe, KVarQ, and PhyResSE, as well as the panel of drugs available in each tool. (DOCX 22 kb)

Additional file 4: **Figure S1.** Distribution of sample numbers in three TB databases. Number of isolates with available DST phenotypes for at least one drug as well as WGS data in the three TB databases PATRIC, ReSeqTB, and LitRev. (DOCX 2339 kb)

Additional file 5: Composition of the dataset analysed in this study. This file presents the details of the databases and studies from which data for the 6756 TB isolates is obtained. (XLSX 115 kb)

Additional file 6: The total number of samples available and the estimated rate of misclassification of phenotypic DST results for each drug. This file presents number of samples with resistance status among the 6756 TB isolates for each drug and the estimated rate of misclassification estimated from the latent class model. (XLSX 9 kb)

## Abbreviations

AMK: Amikacin
CAP: Capreomycin
CIP: Ciprofloxacin
DR-TB: Drug-resistant TB
DST: Drug susceptibility testing
EMB: Ethambutol
ETO: Ethionamide
INH: Isoniazid
KAN: Kanamycin
MDR-TB: Multidrug-resistant tuberculosis
MFX: Moxifloxacin
MLE: Maximum likelihood estimate
*Mtb*: *Mycobacterium tuberculosis*
OFX: Ofloxacin
PAS: P-aminosalicyclic acid
PTO: Prothionamide
PZA: Pyrazinamide
RIF: Rifampicin
SNP: Single nucleotide polymorphism
STM: Streptomycin
TB: Tuberculosis
WGS: Whole genome sequencing
WHO: World Health Organisation
