# Supplementary material for: Genomic prediction of tuberculosis drug-resistance: benchmarking existing databases and prediction algorithms
Source: BMC Bioinformatics. 2019 Feb 8;20:68. doi: 10.1186/s12859-019-2658-z (PMC6368788; doi:10.1186/s12859-019-2658-z)
Supplement: Supplementary file 2 — Quality screening of sequencing data. This file details the quality control step of the study. (DOCX 26 kb) [file 12859_2019_2658_MOESM2_ESM.docx]

**Additional file 2. Quality screening of sequencing data**

Sequencing data was screened using the Qualimap [1] output followed from the PhyResSE pipeline [2]. The sequence reads were first mapped against the H37Rv reference genome with Burrows-Wheeler Aligner (BWA) [3] version 0.7.4. The output was then converted to bam files, sorted and indexed with SAMtools [4] version 0.1.19. Quality of the bam files were then checked with Qualimap [1] version 0.7.1. Any set of raw sequence that yielded a bam file with a mean coverage smaller than 5 was discarded.

***Summary statistics of the mean coverage of the available sequencing data***

| **Min** | **First Quartile** | **Median** | **Third Quartile** | **Max** |
| --- | --- | --- | --- | --- |
| 0.02 | 58.73 | 94.18 | 132.37 | 3052.18 |

***List of isolates filtered***

|  | Isolate | Run accession | Mean coverage | Database |
| --- | --- | --- | --- | --- |
| 1 | SRR1166339, SRR1169098 | SRR1166339 | 2.30 | PATRIC |
|  |  | SRR1169098 | 2.37 |  |
| 2 | SRR1166184, SRR1169012 | SRR1166184 | 0.85 | PATRIC |
|  |  | SRR1169012 | 0.85 |  |
| 3 | SRR1166333, SRR1169104 | SRR1166333 | 1.10 | PATRIC |
|  |  | SRR1169104 | 1.15 |  |
| 4 | SRR3675240 | SRR3675240 | 3.42 | LitRev |
| 5 | SRR3675254 | SRR3675254 | 0.03 | LitRev |
| 6 | SRR3675316 | SRR3675316 | 0.07 | LitRev |
| 7 | SRR3675317 | SRR3675317 | 0.02 | LitRev |
| 8 | SRR3675480 | SRR3675480 | 0.05 | LitRev |
| 9 | SRR3675528 | SRR3675528 | 0.60 | LitRev |
| 10 | SRR3675546 | SRR3675546 | 3.48 | LitRev |

**REFERENCES**

1. Garcia-Alcalde F, Okonechnikov K, Carbonell J, Cruz LM, Gotz S, Tarazona S et al. Qualimap: evaluating next-generation sequencing alignment data. Bioinformatics. 2012;28(20):2678-9. doi:10.1093/bioinformatics/bts503.

2. Feuerriegel S, Schleusener V, Beckert P, Kohl TA, Miotto P, Cirillo DM et al. PhyResSE: a Web Tool Delineating Mycobacterium tuberculosis Antibiotic Resistance and Lineage from Whole-Genome Sequencing Data. J Clin Microbiol. 2015;53(6):1908-14. doi:10.1128/JCM.00025-15.

3. Li H. Aligning sequence reads, clone sequences and assembly contigs with BWA-MEM. arXiv:13033997v2 [q-bioGN]. 2013.

4. Li H, Handsaker B, Wysoker A, Fennell T, Ruan J, Homer N et al. The Sequence Alignment/Map format and SAMtools. Bioinformatics. 2009;25(16):2078-9. doi:10.1093/bioinformatics/btp352.
