# Supplementary material for: Genomic prediction of tuberculosis drug-resistance: benchmarking existing databases and prediction algorithms
Source: BMC Bioinformatics. 2019 Feb 8;20:68. doi: 10.1186/s12859-019-2658-z (PMC6368788; doi:10.1186/s12859-019-2658-z)
Supplement: Supplementary file 3 — Characteristics of the four bioinformatics software for predicting drug resistance from Mtb genome sequences. This file delineates the features of the four tools TBProfiler, MyKrobe, KVarQ, and PhyResSE, as well as the panel of drugs available in each tool. (DOCX 22 kb) [file 12859_2019_2658_MOESM3_ESM.docx]

### Additional file 3. Characteristics of the four bioinformatics software for predicting drug resistance from *Mtb* genome sequences

|  | TBProfiler | MyKrobe | KVarQ | PhyResSE |
| --- | --- | --- | --- | --- |
| Types of data | Fastq; bam | Fastq; bam | Fastq | Fastq |
| User interfaces available | Web; Command line | Command line; Desktop apps | Command line;  Desktop apps (GUI) | Web; Pipelines documentations |
| TB identifications | No | Yes | No | No |
| Basis of resistance prediction | SNPs and indels | SNPs | SNPs | SNPs and indels |
| Sample quality report? | No | No | No | Yes |
| Drugs   \| Isoniazid \| \| --- \| \| Rifampicin \| \| Pyrazinamide \| \| Ethambutol \| \| Streptomycin \| \| Amikacin \| \| Kanamycin \| \| Capreomycin \| \| Quinolones/Fluoroquinolones \| \| Ethionamide \| \| PAS \| \| Aminoglycosides \| \| Bedaquiline \| \| Clofazimine \| \| Linezolid \| | \| X \| \| --- \| \| X \| \| X \| \| X \| \| X \| \| X \| \| X \| \| X \| \| X \| \| X \| \| X \| \| X \| \| X \| \| X \| \| X \| | \| X \| \| --- \| \| X \| \| X \| \| X \| \| X \| \| X \| \| X \| \| X \| \| X \| \| O \| \| O \| \| O \| \| O \| \| O \| \| O \| | \| X \| \| --- \| \| X \| \| X \| \| X \| \| X \| \| X \| \| X \| \| O \| \| X \| \| O \| \| O \| \| O \| \| O \| \| O \| \| O \| | \| X \| \| --- \| \| X \| \| X \| \| X \| \| X \| \| X \| \| X \| \| X \| \| X \| \| X \| \| X \| \| O \| \| O \| \| O \| \| O \| |

For KVarQ, Kanamycin and Amikacin were predicted as one entity.
