# Supplementary material for: Genomic prediction of tuberculosis drug-resistance: benchmarking existing databases and prediction algorithms
Source: BMC Bioinformatics. 2019 Feb 8;20:68. doi: 10.1186/s12859-019-2658-z (PMC6368788; doi:10.1186/s12859-019-2658-z)

**Figure S1. Distribution of sample numbers in three TB databases**

Number of isolates that are distributed in the three TB databases: PATRIC, ReSeqTB, and a set generated from a PUBMED literature review which contributed an additional 455 isolates not present in PATRIC and ReSeqTB. The numbers displayed here represent the number of samples with both WGS data and DST phenotypes for at least one drug.


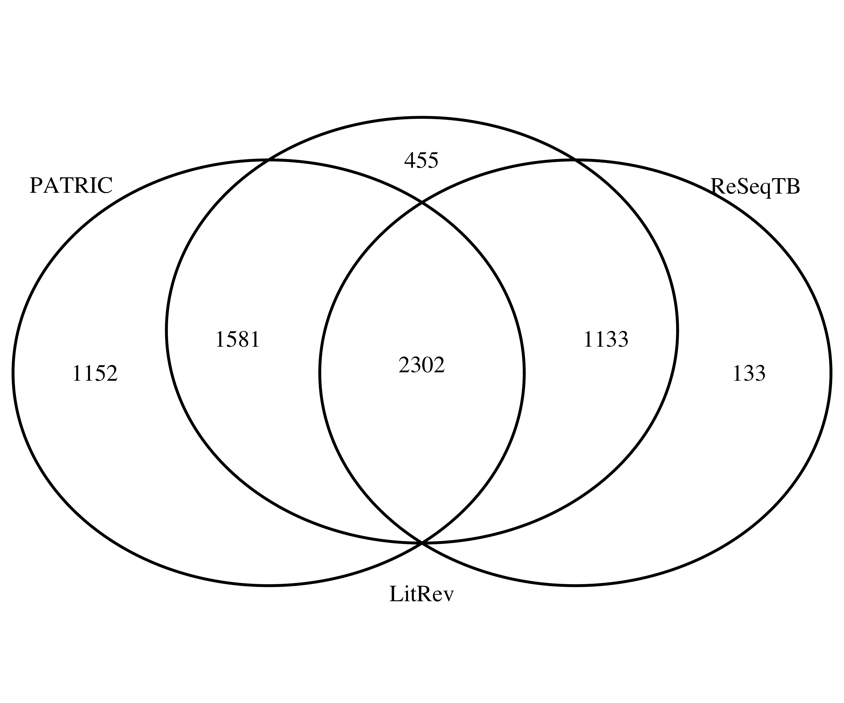

Supplement: Supplementary file 4 — Figure S1. Distribution of sample numbers in three TB databases. Number of isolates with available DST phenotypes for at least one drug as well as WGS data in the three TB databases PATRIC, ReSeqTB, and LitRev. (DOCX 2339 kb) [file 12859_2019_2658_MOESM4_ESM.docx]
